# Facing Muscular Dystrophy During Covid-19 Pandemic: The Role of Support Associations and Spirituality

**DOI:** 10.1007/s11089-022-00997-2

**Published:** 2022-02-03

**Authors:** Lorenza Palazzo, Sara Pompele, Marta Rossi, Gabriella Rossi, Simona Spinoglio, Ines Testoni

**Affiliations:** 1 Department of Philosophy, Sociology, Pedagogy and Applied Psychology (FISPPA), University of Padova, 35131 Padova, Italy; 2 Unione Italiana Lotta Alla Distrofia Muscolare (UILDM) - “G. Bergna” section of Monza and Brianza, 20900 Monza e Brianza (MB), Italy; 3 Emili Sagol Creative Arts Therapies Research Center, University of Haifa, 3498838 Haifa, Israel

**Keywords:** Muscular Dystrophy, Covid-19 pandemic, Associations, Spirituality, Qualitative study

## Abstract

Several researches in scientific literature analyze the theme of Muscular Dystrophy (MD), As well as many others focus on the theme of the Covid-19 pandemic; however, there is a rather limited number of studies that analyse how the pandemic has affected the life of people suffering from MD, especially during the time of the first lockdown in the spring of 2020. The present study has applied a qualitative research design with the aim to investigate how patients with MD have lived the social restrictions imposed for the contagion containment and whether the assistance of associations for their support has contributed to make the participants feel closer or more distant from the spiritual dimension. The analysis involved 12 participants, and they were presented with a semi-structured interview. The data obtained from the interviews have been analysed through a thematic analysis from which 4 thematic areas have emerged: (1) the impact of the pandemic on an emotional level; (2) the illness management and the role of family; (3) the role of the associations; (4) aspects related to spirituality. The crucial role that the closeness of family and the activities promoted remotely by the associations for patients’ support has emerged, since they have allowed the participants to feel united by something beyond, to discover new aspects of themselves, to give more value to Life and to move closer to their spiritual dimension.

## Muscular dystrophy

Muscular Dystrophy (MD) is a recessive illness linked to the X chromosome and it is characterized by a progressive loss of functional muscular mass and by the consequent substitution of fibrous tissue, which contributes to create an association with different neuromuscular disorders and progressive muscular weakness (Sussman, 2002; Yilmaz & Sechtem, 2012). The muscular weakness targets particular muscular groups, which are specifics for the respective genetic illness. The most common forms of MD are the Duchenne Muscular Dystrophy (DMD) – which is the most severe- and the Becker Muscular Dystrophy (BMD) which is a milder kind of dystrophy with a late onset and a slower progression (Goyenvalle et al., 2011). The DMD occurs as a consequence of mutations in the gene that codifies for the dystrophin protein, which leads to the absence or to a defect in such a protein that results in a progressive muscular degeneration (Bushby et al., 2009). 

Currently, the average age during which a person receives a diagnosis is included between 4 and 5 years of age, when these children’s physical ability significantly diverges from the one of their peers, event thought sometimes the first symptoms are noticed at 2 years of age or even before (Ross & Clarke, 2017). The symptoms of the onset of the MD are difficulty in climbing the stairs, a wobbly pace and frequent falls, starting to walk late, walking on tiptoes, a scarce balance and a late development of speech (Duan et al., 2021). The majority of patients become dependent on a wheelchair at around 10–12 years of age and need assisted ventilation at around 20 years of age. With an adequate cure, life expectancy with MD is between 20 and 40 years of age and death usually happens because of cardiac and/or respiratory failure (Verhaart & Aartsma-Rus, 2019). For now, there is no effective treatment to stop the lethal progression of this disease espite the huge treatment progresses of the last 30 years (Abreu & Waldrop, 2020). Nonetheless, the diagnosis can be reached rapidly and the family and the child can be well supported: people affected by MD can reach their full potential in their education and employment (Matthews et al., 2016).

## The impact of the Covid-19 pandemic on patients with previous pathologies

The new coronavirus pandemic (Covid-19) is the current most discussed topic, both within the popular imaginery and the medias, and by the scientific community (Correia et al., 2020). The global incidence of the Covid-19 illness has continued to grow from December 2019, a reason why the 11th of March 2020 the World Health Organization (WHO) has declared the state of global sanitary pandemic (Counted et al., 2020). The symptoms of Covid-19 include fever, coughing, fatigue, shortness of breath, sore throat, headache, diarrhoea and reduced olfactory and gustative sensations. The most serious manifestations, among which there are pneumonia, acute respiratory distress syndrome, cytokine storm, myocardial damage and death, are more common among the older patients and those who present medical comorbidities (Veerapandiyan et al., 2020).

In literature a significant psychological impact of the Covid-19 pandemic has been highlighted: on one hand it has caused some serious threats to people’s physical health and life, but on the other it has also triggered a vast variety of psychological problems, such as panic disorder, anxiety, and depression (Cantelmi et al., 2021; Qiu et al., 2020).

In particular, there is a concern that patients with previous pathologies, such as MD, could be more at risk of developing multisystemic and serious complications in case of Covid-19 positivity due to the numerous of serious comorbidities such as chronic immunosuppression caused by corticosteroids, respiratory insufficiency that leads to the need to a long term ventilatory support and cardiac disfunction (Veerapandiyan et al., 2020). Currently, there are no documented proofs of a higher incidence of coronavirus 2 infection (SARS-CoV-2) linked with severe acute respiratory syndrome among patients with MD; however, the characteristics of the advanced stages of the illness, such as muscular weakness of the thorax or of the diaphragm, kyphoscoliosis, altered cough reflex and clearance of the respiratory tract (unblocking of the respiratory tract), deterioration of the cardiac function, obesity and the steroids intake can predispose to a severe form of the Covid-19 illness (Sobierajska-Rek et al., 2020). The pandemic, moreover, has caused the interruption of many rehabilitation services making the patients with MD run the additional risk of a deterioration of their functional state (Stratton et al., 2020). Moreover, the social isolation associated to the quarantine can also act as a catalyst of many anomalies in mental health, such as acute stress disorders, irritability, insomnia, emotional stress, and mood disorders. A prolonged distance with an elevated level of stress can induce depressive symptoms, panic, and anxiety: this risk appears to be higher for people affected by MD and for their families (Birnkrant et al., 2018; Usher et al., 2020).

## The role of spirituality in the management of a neurodegenerative illness with a poor prognosis

The spiritual dimension is a significant component of the experience of a chronic degenerative illness. Inside the spiritual wellbeing two distinct dimensions can be considered: one exclusively religious, referred to the relationship with God or with what is considered a spiritual being, and a more existential one, which implies a feeling of hope and meaning in life, separated from specifically religious aspects (Unterrainer et al., 2014). Religion and spirituality can be considered multidimensional constructs that interact in various ways with different aspects of the health of a person affected by a chronic illness: they prove to be some real coping strategies that are very often implemented in facing the several challenges of the illness and they are associated with lower psychophysical anxiety and less avoidance strategies (Koenig et al., 2001; Testoni et al., 2016a, b). The effectiveness of the religious and/or spiritual dimension depends on many variables, such as the type of religion, the personal criteria for defining wellbeing, the social context, the individual’s characteristics, and the integration of spiritual elements in the person’s life (Roger & Hatala, 2017). Spiritual wellbeing reflects the transcendent relationship of the single person with a superior being and the experiences he/she has with it through beliefs, values, lifestyle, quality of life and the interactions with oneself, the others and nature (Dal Bello-Haas et al., 2000). The growing relevance of this component in the wellbeing and quality of life of the individual with chronic and degenerative illness with a poor prognosis has led it to enter among Palliative Care practices as a legitimate need that has to be addressed (Unantenne et al., 2011). Moreover, in the field of incurable illnesses, it is more and more evident how healing is distinct from curing, in the common use of the term, since the latter includes a meaning of health and wellbeing consequent to hope, love, relationship with others, finding meaning and purpose in life and illness, and awareness that something bigger than oneself exists (Pehler & Craft-Rosenberg, 2009). Among the positive outcomes on the health of an ill person with a poor prognosis connected to religion and spirituality a deeper contact with meaning and purpose in one’s life and a higher feeling of hope have emerged. Moreover, spirituality improves patients’ life also by reducing the levels of anxiety and depression an ill person can experience: taking care of a person’s spiritual dimension, therefore, brings with it better indicators of health and quality of life (Sytsma et al., 2020). A certain spiritual wellbeing in people with chronic illnesses significantly predicts patients’ satisfaction towards their life and their acceptance of their physical state (Chen & Crewe, 2009). In the case of patients with a neurodegenerative illness with a poor prognosis it has been observed that giving a meaning to their life helps them give a meaning to their illness as well, to find coherence and meaning to the experience they are facing (Dal Bello-Haas et al., 2000; Faull & Hills, 2006). Indeed, a person with high levels of existential wellbeing, even if not religious, can experiment high levels of spirituality; a religious belief can be helpful, however it does not appear to be necessary in order to achieve satisfaction from a spiritual point of view (Pagnini et al., 2011). Spiritual closeness of patients with chronic neurodegenerative illnesses has a positive impact also on caregivers’ mental health, who, as it has been highlighted in literature for many years, present high levels of depression, stress and a lower quality of life compared to patients’ ones (Gauthier et al., 2007; Trail et al., 2003). If the patient finds a resource in spirituality that brings a higher meaning to his/her life and more inner strength, the psychological impact of his/her illness on people closer to him/her could decrease (Pagnini et al., 2011).

## Methods

### Aims

The first purpose of the present study was to explore the experiences of people affected by MD during the Covid-19 pandemic especially during the lockdown period of the spring in 2020. More specifically, the aim was to observe how the different restrictions, implemented to contain the spread of the virus, have impacted their life and the management of their illness.

Secondly, another aim was to observe what kind of role participants’ spirituality had in facing the period of closures and restrictions and whether the closeness and support of the associations has contributed to the grow of intimacy of participants to their spiritual dimension.

### Participants and data collection

The research project has involved 12 participants affected by neuromuscular illnesses, more specifically 9 people affected by MD, 2 people with a non-specified neuromuscular pathology and 1 person affected by a type 2 Spinal Muscular Atrophy. The group was composed of 9 males (75%) and 3 females (25%) of an age between 23 and 65 years (M = 37.8; SD = 13.8). Concerning their educational degree, 5 (42%) had a university degree, 5 (42%) had graduated at high school, and 2 (17%) had a middle school licence. As regards their employment at the time of the interview, 2 (17%) were still students at a university master’s degree, 4 (33.3%) had a job, 5 (42%) were unemployed and 1 (8.33%) was retired. Lastly, concerning their religious beliefs, 6 (50%) participants declared to be Catholics, 3 (25%) declared to believe in a personal spiritual dimension independent from a religion and 3 (25%) defined themselves as non-believers. Table 1 summarises participants’ biographical information and their age when they received their diagnosis. The reported names are fictional to avoid the possibility to recognize the participants. An informed consent was obtained by all participants before they took part in the study. The study followed the American Psychological Association’s Ethical Principles of Psychologists and Code of Conduct and the principles of the Declaration of Helsinki. It received research ethics approval from the Health Sciences and Science Research Ethics Committee of the University of Padova (reference: C3DD8C5FCE1C26C7E80954B4EC34DC16).

**Table 1:** Participants’ demographic characteristics

**Fictitious names**	**Age**	**Sex**	**Educational level**	**Occupation**	**Age at diagnosis**
Marco	23	M	Bachelor's degree in linguistic mediation	He is waiting to start his master's specialization	3
Alessandro	25	M	Bachelor's degree in legal services sciences	Unemployed	At birth
Cristiano	42	M	Middle school diploma	Unemployed	13
Mattia	23	M	Scientific high school diploma	Student of sciences of cultural heritage at the state University of Milan	5/6
Laura	37	F	Bachelor's degree in law	Unemployed	13
Paola	56	F	Middle school diploma	Retired	12/13
Fabio	42	M	High school diploma	Unemployed	4/5
Michele	50	M	Degree in electronic engineering	Employee in a company in the telecommunications field	44
Antonio	65	M	Accounting school diploma	Freelancer in condominium administrations	10
Guido	24	M	Diploma in administration, finance and marketing	Unemployed	20 months
Salvatore	30	M	High school diploma	/	3
Serena	37	F	Master's degree	Educator, counselor and psychologist	6 months

Participants have been contacted through the Italian national association of reference for MD, the “Unione per la Lotta alla Distrofia Muscolare” (UILDM) (Union for the Fight against Muscular Dystrophy), which conducts researches, assists people with neuromuscular illnesses and has the objective to facilitate the social inclusion of people with disability, to promote scientific research and sanitary information on MD and other neuromuscular pathologies (UILDM, 2017). Participants were illustrated the research aims and they were requested to sign the informed consent for the participation in the study and for the audio-recording of the semi-structured interview before its start. The interview has been administered by a researcher and counsellor of the research team. The interviews lasted approximately 30 min and each interview has been audio-recorded and subsequently transcribed verbatim. The interviews investigated the emotions prevalent during the lockdown period, the impact of the pandemic on the illness management and the eventual difficulties, the potential changes that took place during the lockdown, the possible presence of support from the family, the support received from the associations and whether and how such a assistance had made the participants feel more linked with a greater dimension beyond, and, lastly, whether their religious and/or spiritual belief had increased or decreased in the pandemic period.

### The thematic analysis

The analysis of the semi-structured interviews has been carried out through thematic analysis, which is based on the identification of models of meaning or themes inside the qualitative data (Maguire & Delahunt, 2017). The objective of this analysis method is to identify themes, that are models in the data that are important or interesting, and utilizing these themes to address the research or saying something concerning a problem. This is much more than a simple summary of the data, since a good thematic analysis gives meaning to them, making it emerge from the qualitative data themselves (Clarke & Braun, 2013). In particular, in the present research has been used a bottom-up method of qualitative analysis, which aimed not to look for some categories pre-established by the researcher, but instead to make the themes and the meaning of the data emerge from the interviewed participants’ words. The analysis has been conducted by two researchers who, after working on it separately, have compared the emerged results and unified the similar thematic areas; they have proceeded following those steps: familiarizing with the data, creation of codes, research, revision, definition and denomination of the themes, and production of the final report (Braun & Clarke, 2006). Lastly, a third researcher has monitored every step and supervised the final themes that have emerged. The thematic analysis has been conducted with the dedicated software Atlas.ti (Gibbs, 2007).

## Results

The analysis has defined four thematic areas: (1) the impact of the pandemic on an emotional level; (2) the illness management and the role of family; (3) the role of the associations; (4) aspects related to spirituality.

Some of the participants’ answers have been reported as an example for each thematic area, and they have been reformulated in order to avoid the possibility to recognize the interviewees’ identity.

### First thematic area: the impact of the pandemic on an emotional level

The main emotions reported by the participants were sufferance, boredom, sadness, frustration, and rage; for many of them it was challenging to be without their loved ones whom they have greatly missed, as well as the fact of not having the possibility to freely go out of their home or having to renounce to many aspects of their lives since they did not agree with some decisions that have been made during the lockdown. Some also reported the fear of contracting Covid-19 and die or to have to face the serious consequences of the virus together with their condition of illness. For example, Paola said:

The emotion I felt the most was fear, fear of this virus, the world disruption. My fear and anguish were strong. Many say, “It is like this for everyone”, but it is not true, not for me since I am a person with disability, and I live this first-hand always. What worries me the most, scares me the most, more than the thought of the contagion, is the fact of having this pathology. I am scared of suffocating to death, of not breathing anymore.

Others, despite this fear, tried to find a positive aspect, such as the opportunity to take the lockdown moment to cultivate old passions and new interests, find a new balance, developing more autonomy and understanding the value of their life-experiences and existence more deeply. Some said they tried to move from the resentment towards a condition of hope to return soon to normalcy, engaging in different activities to direct their thoughts towards something else rather than their strong concern or boredom. Laura, for example, said:

This moment has taught me to manage on my own, to engage in activities on my own, it taught me to seize the little occasions even without being in a comfortable condition.

Agreeing on this, Mattia explained:

I tried to free myself from this feeling, I started to engage in some activities I had not carried out for many years: for example, I dedicated some time to reading. Since I had a lot of time during the day, more than usual, I tried to organize myself in order to make my days full of different activities.

### Second thematic area: the illness management and the role of family

The management of participants’ disability and the eventual suspension of their treatments—such as physiotherapy and medical examinations—in such an anomalous period was also investigated in the interview. The majority of participants reported they did not have any particular difficulties in the management of their disability during the lockdown, since the presence of assistants had always been constant or because their life was already organized in a way to guarantee them autonomy. However, a small number of participants did encounter some difficulties as Mattia affirmed:

Certainly, one of the most complex aspects was not being able to continue my physiotherapy with the doctor because of the closures, so for sure this was an important problem because, since I am used to have my therapy session once a week, and spending an entire month without doing it negatively impacted by it.

Undoubtedly, with the beginning of the pandemic a significant amount of jobs have been stopped for some months, consequently family life underwent some changes. We have investigated, therefore, how this modification has influenced our MD patients’ life. For the most of them the increased presence of family members represented a great support both from the physical, practical and emotional point of view. Salvatore, for example, said:

Being able to have someone to be together with was certainly positive, if I had been alone, I would have faced more shortcomings, but since I had my family members and my sister I lived the lockdown well, especially from an emotional point of view. They helped me a lot, on an emotional level, we laughed and talked about this and that, but they also helped me in many little practical things.

### Third thematic area: the role of the associations

For many participants being able to have the association close to them, even if in a telematic way, was helpful in alleviating their days and also in making them feel less lonely. Cristiano, for example, said:

We engaged in different activities, for example some games such as treasure hunt through mobile phone, or we sent each other pictures of what we were doing during the days. The association stayed close to us, so I did not miss it. I missed going there every Friday, because that was very nice too, the fact of being together, I missed that above all.

Concerning the same aspect, Michele said:

The activities we conducted remotely were very useful, they contributed to maintain social relationships, which were prohibited face to face, they helped a lot. Even though only virtually, we could still see each other, we could speak with one another, they were nice moments even though they were long-distance ones.

Since the support and closeness often make people feel they are bound to something greater beyond, we have also explored whether the support received from the association had made the participants feel more at peace with Life or more reassured by a superior presence. Regarding this, participants could be divided in two groups: some of them said they undoubtedly felt the association’s closeness, but they did not link it with other aspects beyond that; others, instead, affirmed that the received support made them feel a gratitude and a sense of protection coming from something greater than themselves. For example, Michele said:

The association’s support helped me feel more at peace with Life thanks to the meditation activities and it helped me discover new ways to be creative and to pass the time, especially thanks to the creative writing activities and to the musical-dramatic laboratory. With “higher presence” I mean the one that is inside us, the divine and creative nature we all have, and the association helped us better connect with it.

### Fourth thematic area: aspects related to spirituality

Inside this thematic area have been inserted the participants’ answers related to the theme of spirituality, especially whether and how they had felt closer or more distant from feeling the presence of a superior being, or God for the ones who were believers. Some of them said they did not feel close to any spiritual practice and that they have never been not even before the pandemic; some of them said they addressed their thoughts to God even though they were non-believers, especially during the most difficult times of the lockdown, while others said they felt even more distant from superior Beings. Paola, for example, said:

During the pandemic period, I spent the majority of my time alone, so I had the possibility to reflect a lot. Although I am not a devout and not a believer at all, I spoke to God anyway to ask for help for all the people who had contracted Covid-19 and to ask God to help the world defeat it.

Some participants declared themselves spiritual without embracing a particular religious belief. Concerning what for them could be meant with “Superior Being”, some indicated their loved ones, especially the ones who are not alive anymore, their divine and creative nature, what one feels inside of himself/herself and not on the outside. As regards the definition of a personal concept of spirituality, for example, Laura said:

In my spiritual practice, I am the one who decides what is closer or more distant from me, I do not depend upon something outside myself. Spirituality for me is having inside me the courage, not depending upon a Church, it does not depend on anything outside myself. I am the engine and I only need the fuel of my spiritual practice that allows me to put into practice the things of my life.

For Serena, the lockdown period allowed her to get closer to the spiritual dimension:

This period put me in the condition of having more time, space, and motivation to meditate and connect with Life and with whom accompanies me in other dimensions. What life puts on my path, such as, in this case, the support I received, generates in me gratitude and a sense of protection and care towards myself from something greater than me.

Similarly, to Serena, Marco lived the pandemic period as an occasion to feel even closer to something superior as well:

The pandemic has been an occasion to connect with the superior being inside of us, with our spirituality, rediscovering the value of silence, and learning to feel better with ourselves, to discover new forms of creativity, to meditate.

## Discussion

The present study had the aim to investigate the experiences lived by people affected by MD during the Covid-19 pandemic, specifically during the toughest period passed in Italy during the lockdown in the spring of 2020, and to observe how this period and its restrictions had impacted on these patients’ quality of life and illness management. Secondly, the research wanted to observe the role of spirituality during the very same period and, in particular, whether the closeness and the support of the associations had contributed to make the participants feel closer to the spiritual dimension.

The spread of the new Covid-19 epidemics, like other epidemics, has caused psychological, social and physiological stress (Khasawneh, 2020). The state of stress could be temporary, when the individual suffers for a brief period and then the stress ends without leaving any long-term effects, but it could be long-term in the case of people with disabilities who suffer from illnesses and chronic health problems (Groce, 2005). From the analysis of the interviews, it has emerged that the main emotions experienced by participants have been sufferance, boredom and sadness for not being able to go out and frustration since they could not choose to leave their home freely. These results are in line with what has already been observed in literature: Covid-19 not only had an impact on the health of people with disabilities, but it also had an impact on psychological, social and economic aspects of this category of individuals (UNSDG, 2020). As with our participants, some consequences reported in literature by patients affected by neuromuscular illnesses and by their family members during quarantine have been an increase of their anxiety and feelings of loneliness (Consonni et al., 2020). Given their previous condition of illness, these people are indeed more susceptible to psychological problematics or to have underlying conditions of mental health in comorbidity with MD that increase their vulnerability (Summaka et al., 2021). Consequently, they had to face greater difficulties in the development and implementation of appropriate coping strategies (Kar et al., 2020).

The present Covid-19 epidemics has imposed new security measures and restrictions that have frustrated the different populations around the whole world (Summaka et al., 2021). The problematics linked with lack of sport, movement and physical activity encountered in the participants’ declarations are in line with the existing literature. The containment measures could have had some adverse effects on the population health: quarantine has indeed entailed an abrupt change in people’s lifestyle, leading to an increase in sedentary behaviour (Maugeri et al., 2020). Such a radical change in daily life can have negative effects on high-risk patients, who must practice regular physical exercise to contrast the negative consequences of certain illnesses, such as neuromuscular ones (Vetrovsky et al., 2020). Isolation at home can lead to an unhealthy diet, low quality of sleep, reduced levels of physical activity and to sedentary habits with several negative consequences, such as an increase in body fat, decrease in muscular mass, insomnia and depression. Moreover, the entity of the reduction in physical activity is correlated with the perceived physical and mental health of people with neuromuscular illnesses, influencing therefore their quality of life (Di Stefano et al., 2020).

Concerning the management of disability, in literature we can find that people with neuromuscular illnesses experience different problems: for example, Mauri et al. (2020) observed that auxiliary services such as rehabilitation, home-based nursing assistance and psychological support have been significantly affected on a national level. Because of the additional risk of infection related to the close patient-therapist contact, the rehabilitation services have generally been suspended (Natera-de Benito et al., 2021). In contrast with what has just been reported, the participants of this research affirmed they have not encountered specific difficulties in the management of their illness from the point of view of the assistance, since the presence of assistants remained constant during the whole quarantine; only some participants have complained about some problems concerning physiotherapy: this aspect is in line with the existent literature (Gitkind et al., 2020). Many patients affected by neuromuscular illnesses have suffered home isolation and the temporary interruption of a part of their medical appointments and ambulatory exams, finding it difficult to receive standard therapies and rehabilitation sessions and facing the risk to see a worsening of their illness or an exacerbation of their symptoms (Bertran Recasens & Rubio, 2020; Tseng & Chen, 2021).

Lastly, concerning the second aim of the present study, it has been observed that the role of the association has been substantial for the participants, who have highlighted the importance of being able to participate in some activities and feeling close to one another, despite the inevitable lack of physical presence. Some participants have perceived this support as something that has allowed them to feel at peace with Life and reassured by the presence of something greater beyond with which they could connect thanks to their feelings of hope, gratitude and creativity experienced during the proposed activities, and, as has been observed in literature, linked with the good quality of care and associated to a better quality of life (Faull & Hills, 2006; Pehler & Craft-Rosenberg, 2009). Some answers received by the participants and inserted in the fourth thematic area allowed us to confirm what had already been observed in literature, that is, the meaningful role of spirituality in making these patients feel stronger and in allowing them to feel inside of themselves the courage to face both the illness and the difficulties linked to the pandemic (Chen & Crewe, 2009; Sytsma et al., 2020). The study also had the interest to observe whether during the lockdown period people had gotten closer or more distant from the spiritual dimension; only three participants have defined themselves as non-believers inside our group: two of them remained in this position, while instead one of them has felt the necessity to address a superior Entity to ask for help in a difficult moment such as the one faced the previous year. Among the ones who already declared to be believers or close to a spiritual practice, the meditation and expressive activities, but also the virtual socialization games, have allowed them to connect with something beyond greater than themselves; these activities, together with the increased time to dedicate to themselves and Life, have allowed them to feel closer to spirituality and to be able to see an occasion and a possibility to discover new aspects of themselves, to rediscover aspects of life that had been taken for granted and to appreciate their value, even in the difficulties of the illness and of the pandemic (Dal Bello-Haas et al., 2000; Testoni et al., 2016a, b, 2019).

## Conclusions

The present study allowed to understand the reality of people who face an already complex illness such as MD during the additional difficult situation faced on a global level because of the Covid-19 pandemic. It was possible to observe which emotions have been the most relevant, how they have been managed and what have been the main sources of support. Moreover, the study also allowed to observe how the support received from the associations made the participants feel closer to the spiritual dimension, which is considered a predictive factor of a good quality of life and of increased strength, courage, hope and wellbeing when one must face a condition of incurability. The importance of this research resides, indeed, in the enrichment it has offered to this important theme: the present study has shed light on a theme that is scarcely explored in literature, that is, the psychological impact of Covid-19, of the pandemic and of all the consequent restrictions on people affected by serious illnesses, MD in particular.

## Limitations and future developments

The research presents some limitations, in particular the limited variety of studies linked with this theme and consequently the impossibility to adequately compare it with the existent literature. Moreover, a second limitation of the research is the small number of participants who could be contacted to take part in the research.

Future studies could broaden the research by taking into consideration a larger sample of participants who suffer of different types of neurodegenerative illnesses and other pathologies, comparing them with one another based on their peculiarities.

## Data Availability

The datasets used and/or analysed during the current study are available from the corresponding author on reasonable request.
